# Design and Immunogenicity of SARS-CoV-2 DNA Vaccine Encoding RBD-PVXCP Fusion Protein

**DOI:** 10.3390/vaccines11061014

**Published:** 2023-05-23

**Authors:** Dmitri Dormeshkin, Mikalai Katsin, Maria Stegantseva, Sergey Golenchenko, Michail Shapira, Simon Dubovik, Dzmitry Lutskovich, Anton Kavaleuski, Alexander Meleshko

**Affiliations:** 1 Institute of Bioorganic Chemistry of the National Academy of Sciences of Belarus, 220084 Minsk, Belarus; 2 Immunofusion, LLC, 210004 Vitebsk, Belarus; 3 Imunovakcina, UAB, LT-08102 Vilnius, Lithuania; 4 Institute of Science and Technology Austria, 3400 Klosterneuburg, Austria

**Keywords:** COVID-19, SARS-CoV-2, DNA vaccine, receptor binding domain

## Abstract

The potential of immune-evasive mutation accumulation in the SARS-CoV-2 virus has led to its rapid spread, causing over 600 million confirmed cases and more than 6.5 million confirmed deaths. The huge demand for the rapid development and deployment of low-cost and effective vaccines against emerging variants has renewed interest in DNA vaccine technology. Here, we report the rapid generation and immunological evaluation of novel DNA vaccine candidates against the Wuhan-Hu-1 and Omicron variants based on the RBD protein fused with the Potato virus X coat protein (PVXCP). The delivery of DNA vaccines using electroporation in a two-dose regimen induced high-antibody titers and profound cellular responses in mice. The antibody titers induced against the Omicron variant of the vaccine were sufficient for effective protection against both Omicron and Wuhan-Hu-1 virus infections. The PVXCP protein in the vaccine construct shifted the immune response to the favorable Th1-like type and provided the oligomerization of RBD-PVXCP protein. Naked DNA delivery by needle-free injection allowed us to achieve antibody titers comparable with mRNA-LNP delivery in rabbits. These data identify the RBD-PVXCP DNA vaccine platform as a promising solution for robust and effective SARS-CoV-2 protection, supporting further translational study.

## 1. Introduction

The current ongoing global pandemic caused by severe acute respiratory syndrome coronavirus 2 (SARS-CoV-2) has resulted in more than 6.5 million coronavirus disease (COVID-19)-related deaths as of September 2022 (www.worldometers.info/coronavirus/, accessed on 10 July 2022).

SARS-CoV-2 is a new pathogenic human coronavirus, which also includes hCoV-OC43, hCoV-HKU1, hCoV-229E, hCoVNL63, and two life-threatening beta-coronaviruses: Middle East respiratory syndrome coronavirus (MERS-CoV) and SARS-CoV [1]. The viral genome is represented by a positive-sense, single-stranded RNA encoding four major structural proteins: spike (S) in the form of a homotrimer, nucleocapsid (N), membrane (M), and envelope (E) [2]. S proteins are cleaved at a polybasic cleavage site at their S1/S2 junctions into S1 and S2 subunits during their biosynthesis in infected cells by proprotein convertases, such as furin. The S protein of the mature virion consists of these two non-covalently associated subunits [3].

The entry of SARS-CoV-2 begins with the binding of receptor binding protein (RBD) within the S1 subunit of the S protein to angiotensin-converting enzyme 2 (ACE2) on a host cell, leading to conformational changes, S1 shedding, and an additional S2 cleavage site exposition. Later, transmembrane protease serine 2 (TMPRSS2) at the cell surface or cathepsin L in the endosomal compartment operates cleavage at the S2 site, releasing the fusion peptide and subsequently initiating a fusion pore formation [4]. The position switching of the RBD from down to up executes receptor binding, while up-to-down switching assists the virus in impeding immune surveillance. The availability of the receptor binding-determining region to the ACE2 is controlled by the hinge-like conformational motion of the RBD [5]. A mutation at the 614 amino acid, i.e., Asp614-Gly, being present in all mutant strains and absent in the wild-type Wuhan-Hu-1 strain has been reported to enhance the up-conformation of the RBD and diverse epitopes accessibility. Such configuration makes the virus more infectious and susceptible to neutralizing antibodies [6]. The last fact could be taken into account in S-protein-based SARS-CoV-2 vaccine development, because closed S proteins could expose other conformational epitopes, eliciting different antibody-specificity responses [7].

N and M proteins, as well as Nsp8 and ORF9b of SARSCoV-2, are engaged in escape from detection by innate immune sensors. The SARS-CoV-2 N protein suppresses the activity of STAT1 and STAT2, interfering with the IFN signaling pathway [8]. Moreover, the M protein is able to bind RIG-I, MDA5, MAVS, and TBK1 to prevent their interaction, suppressing type I and III IFN production [9]. Nsp8 and ORF9b proteins also suppress the IFN signaling pathway by directly binding to MDA5 CARD and the translocator of outer membrane 70, respectively [10,11]. These defects in innate immunity caused by SARS-CoV-2 could impair humoral and cellular immunity acquisition. In one study, it was shown that serum from vaccinated patients had 17-times-higher neutralization capacity than serum collected from patients after natural infection [12].

Effective vaccines have been developed and globally introduced, including inactivated whole-virus vaccines, protein/peptide subunit vaccines, nanoparticle vaccines, vector vaccines, and nucleic-based formulations (mRNA and DNA vaccines), each having its own pros and cons [13,14,15].

Despite the unprecedented success in SARS-CoV-2 vaccine development, incorporating the S protein from wild-type Wuhan-Hu-1 strain, novel strains of SARS-CoV-2 have emerged facilitating mild to moderate escape from humoral immunity, which include, but are not limited to, B.1.1.7 (UK; alpha variant), B.1.351 (SA; beta variant), P.1 (Brazil; gamma variant), B.1.617.2 (India; delta variant), and, recently, Omicron (B.1.1.529) with its derivatives. Several studies reported a 2- to 4-fold reduction in neutralization against Delta, a 6-fold or higher reduction in neutralization against Beta, and a 29-fold reduction in neutralization against Omicron for both convalescent and vaccinated individuals [16,17,18]. Despite reduced vaccine efficacy against mutant strains, effectiveness remained high against hospitalization or severe disease [19].

Omicron has 32 mutations in the S-protein, and 15 mutations are located in the RBD, which is the main key for viral–cell interactions and entry mediated by ACE-2 [20,21]. Serum from vaccinated patients or convalescents from Alpha (B.1.1.7), Beta (B.1.351), and Delta (B.1.617.2) SARS-CoV-2 infection has substantially decreased the neutralization capacity of the Omicron subvariant, as well as therapeutic antibodies [22,23]. An additional prolonged antigen stimulation by a booster vaccine or infection can increase the neutralization breadth and resistance to RBD mutations, for instance, against Omicron, which is reflected by an ongoing germinal center reaction in RBD-specific memory B-cells with somatic hypermutations acquisition, affinity maturation, and, at the same time, contraction of humoral responses to other SARS-CoV-2 domains [24,25,26]. It has been shown that the homotypic nanoparticle RBD SARS-CoV-2 vaccine induces broad cross-reactive neutralization and binding to zoonotic coronaviruses (SHC014, WIV1, Yun 11, BM-4831, and BtKY72), in which RBD sequence homology varies from 68% to 95%. Such results were not reproduced by soluble S-trimeric vaccine and convalescent serum [27].

RBD, NTD, and S2 are the main domains of SARS-CoV-2 that could induce neutralizing antibodies [28,29]. SARS-CoV-2 RBD is the main immunodominant site of the S protein for neutralizing antibodies induction. It has been shown that the depletion of anti-RBD antibodies in convalescent patient sera results in the loss of more than 90% of neutralizing activity of the sera against SARS-CoV-2 [30].

RBD is considered a low-immunogenic antigen due to the relatively short length of the antigen and strategies to promote immunogenicity, including the use of appropriate adjuvants or the addition of exogenous sequences capable of potentiating immune responses should be discussed in vaccine development [31]. Previous studies revealed that vaccines incorporating the SARS-CoV full-length S protein could induce harmful immune responses with enhanced infection or liver damage after virus challenge that are not seen with RBD SARS-CoV vaccines [32,33,34]. Involving the RBD antigen om SARS-CoV-2 vaccine development instead of the full S-protein could potentially decrease the probability of antibody-dependent enhancement (ADE) development, as well as induce stronger humoral and cellular immune responses [35,36,37].

Despite the fact that mRNA vaccines became one of the breakthroughs in COVID-19 vaccine development, DNA vaccines are gaining more and more attention. DNA vaccines have not reached their full potential yet and have a wide window of improvement, including DNA delivery, in vivo expression enhancement, and vaccine design improvement. There are several advantages of DNA vaccines: an unlimited number of reapplications due to the lack of immune response against the vector, natural Th1 response skewing, flexibility in vaccine upgrade, and simplicity and rapidity to make and scale up production with a resultant affordable price for the product. Furthermore, DNA vaccines have been proven to be safe and not reactogenic, possess high stability, and have no need for a sophisticated low-temperature supply chain [38].

The MERS DNA vaccine has been tested in a Phase I trial, where seroconversion occurred in 61 (94%) of 65 participants after two and three vaccinations. T-cell responses were detected in more than 71% percent of patients. Response tended to be stable: at week 60, vaccine-induced humoral and cellular responses were detected in 51 (77%) of 66 participants and 42 (64%) of 66, respectively [39].

Most typically, DNA vaccines yield low immune responses, mainly due to the inefficiency of DNA delivery into macroorganisms. Improved delivery by electroporation and needle-free injectors (for example, the PharmaJet Needle-free Injection System) increased in vivo antigen expression and immune responses to DNA vaccines. Other approaches, such as the reduction of the size of the DNA plasmid, deletion of antibiotic-resistance genes, and the addition of immunostimulatory sequences into the backbone of the DNA vaccine has been shown to increase the immunogenicity of the SARS-CoV-2 DNA vaccine [40].

However, there is an additional window for DNA vaccine improvement [41,42,43]. To deepen the humoral immune response to our DNA SARS-CoV-2 vaccine, we implemented several solutions: (I) fusion of the SARS-CoV-2 antigen to Potato virus X coat protein (PVXCP) to ensure the assembly of VLP and Th1 immune response skewing: (II) selection of RBD as an antigen of SARS-CoV-2 to enhance protection from VOCs and ensure the safety of our SARS-CoV-2 vaccine.

Based on the aforementioned arguments, we developed different designs of SARS-CoV-2 DNA vaccines incorporating RBD as a backbone antigen and compared their efficacy in vivo. The plasmid DNA vaccine candidate encoding Omicron BA.1 SARS-CoV-2 RBD and PVXCP demonstrated a high antibody response and a strong Th1-biased cellular response. It was demonstrated that the delivery of the DNA vaccine RBD-PVXCP by the needle-free injection system induced >300,000 RBD-specific IgG mean endpoint titer in rabbits. Our results thus identify RBD-PVXCP as a robust and effective vaccine candidate against SARS-CoV-2.

## 2. Materials and Methods

### 2.1. Molecular Dynamics Simulation

The spatial structures of the RBD and HR2 domains were obtained from PDB (PDB IDs: 7KLW and 6XR8 respectively), as well as the peculiarities of the RBD-HR2 domain connection [44,45]. For the construction of the 3-dimensional structure of PVXCP, the PDB structure with PDB ID 6R7G was used [46]. Three protein domains were connected into one chimeric protein structure, taking into account the allowed values for φ and ψ angles (various resulting structures were additionally checked using the Molecular Modelling Toolkit python library).

For further structure optimization of chimeric protein molecular dynamics (MD), the minimization, heating, equilibration, and free dynamics stages were performed. The minimization protocol included 4500 steps using the gradient descent method and 500 steps using the conjugate gradient method. Heating was carried out for 1 ns to a temperature of 298.15 K (NVT ensemble). In the next step, the simulated system was equilibrated for 1 ns at 298.15 K and constant pressure. The simulation of free dynamics was carried out with a time interval of 500 ns (NPT ensemble). Constant pressure in the system was maintained using an external barostat (relaxation time of 2 ps). A constant temperature was maintained using a Langevin thermostat (collision frequency of 2 ps–1). At all stages of modeling, the cutoff value was equal to 8.0 Å. The calculation was carried out in an explicit solvent (water, TIP3P model, and the size of the modeling area was 8.0 Å from the protein surface).

After MD, the optimized structure of the chimeric molecule RBD-HR2-PVXCP was used to replace the original monomers of the structure from 6R7G (one helix turn). The obtained multimolecular complexes were analyzed by the previously described MD (50 ns simulation) in order to estimate the probability of complex dissociation.

### 2.2. Preparation of DNA Constructs

Codon-optimized synthetic sequences were generated by gene synthesis (Synbio Technologies, Monmouth Junction, NJ, USA) and subcloned into a mammalian expression vector pIF (Figure S2). This vector was designed for in vivo DNA delivery and had a reduced backbone size (3.1 kb) with a minimal set of bacteria-originated elements, including the pBR322 origin of replication, kanamycin resistance gene, CMV enhancer/promoter without intron A, and bGH poly(A) signal. Transfection-grade plasmid DNA was isolated from an *Escherichia coli* XL10 Gold (#200314, Agilent, Santa Clara, CA, USA) cell overnight culture with the NucleoBond™ Xtra Midi Kit (Macherey-Nagel, Düren, Germany).

### 2.3. In Vitro DNA Vaccines Expression

All the plasmids for analytical expression were purified using a PureLink™ HiPure Plasmid Miniprep Kit (#K210003, Invitrogen, Waltham, MA, USA). Plasmid vectors were transiently transfected into FreeStyle™ 293-F cells (#R79007, Gibco, Billings, MT, USA) using polyethylenimine (PEI) transfection. Twenty-four hours before transfection, 293-F cells were subcultured to a final density of 5 × 10^5^ cells per milliliter of antibiotic-free FreeStyle medium to reach 1 × 10^6^ cells per milliliter by the transfection day in a total volume of 2 mL. DNA and PEI were taken in amounts of 2 μg and 3.2 μg, respectively, per well. DNA:PEI complexes were produced by mixing separately prepared solutions of 2 μg of DNA and 3.2 μg of PEI diluted by OptiMem medium to a final volume of 37 μL. After adding PEI solution to DNA, the mixture rested at room temperature for 25 min. The final mixture was added to the cells, followed by placing them in an incubator (37 °C and 5% CO_2_) for 5 days with constant shaking of 120 rpm. The conditioned expression medium was centrifuged to remove cell debris, and then the cell medium was analyzed by Western Blotting using anti-S-protein polyclonal antibodies (#E-AB-V1006, Elabscience, Houston, TX, USA). The expression levels of v1 and v2 were assessed by comparison with the v0 expression level by Western Blotting. The expression level of v0 (5 ng per 1 μL of expression medium) was measured after the recombinant RBD purification and quantification.

### 2.4. Identification of SARS-CoV-2 Binders

Tris–glycine Novex gels (#EC6021BOX, ThermoFisher Scientific, Waltham, MA, USA) were loaded with 10 μL of cell supernatant on the 5th day after transfection. Gels were run at 120 V for 1.5 h in Tris–tricine–SDS buffer. After electrotransfer in the Towbin buffer using a Trans-Blot Turbo Transfer System (Bio-Rad, Hercules, CA, USA), the PVDF membrane was blocked with 5% filtered skimmed milk in PBS overnight at 4 °C. The membrane was washed and then incubated with 1:5000 SARS-CoV-2 Spike RBD Polyclonal Antibody antibodies (#E-AB-V1006, Elabscience) for 1 h at RT and with 1:10,000 Goat Anti-Rabbit-HRP (#31460, Invitrogen) for 1 h at RT. After washing, the membrane was developed using ECL Clarity Substrate (Bio-Rad, USA) and imaged using Azure C300 imager (Azure Biosystems, Dublin, CA, USA).

### 2.5. Mice Immunization

BALB/c female mice (6 weeks old) were purchased from the Rappolovo Animal Farm of the Russian Academy of Medical Sciences and housed in the animal facility of the Institute of Physiology of the National Academy of Sciences of Belarus (Minsk, Belarus). For vaccination purposes, the mice received a 50 μL intramuscular (IM) injection of 2.5 μg or 50 μg of pDNA with the subsequent electroporation (EP) in the tibialis muscle of the shaved hind leg on days 0 and 14 of the experiment. EP was performed with an AgilePulse In Vivo System (BTX). Prior to immunization, all of the mice were anesthetized by isoflurane inhalation with RAS-4 Rodent Anesthesia System (PerkinElmer, Waltham, MA, USA). Mice were euthanized on day 28 for terminal blood collection and their spleens were harvested for cellular assays. All animal experiments were conducted according to the Belarusian Guide for the Care and Use of Laboratory Animals.

### 2.6. Rabbits Immunization

Ten female New Zealand white rabbits were housed in the animal facility of the Institute of bioorganic chemistry of the National Academy of Sciences of Belarus (Minsk, Belarus). The rabbits were divided into four groups:

- Group 1 (*n* = 2): 100 μg pDNA (vector control—pIF) solution in PBS was administered intramuscularly by needle injection;
- Group 2 (*n* = 2): 100 μg pDNA (v1) solution in PBS was administered by intradermal needle injection followed by electroporation with an AgilePulse In Vivo System (BTX);
- Group 3 (*n* = 3): 100 μg pDNA (v1) solution in PBS was administered intramuscularly by needle injection;
- Group 4 (*n* = 3): 100 μg pDNA (v1) solution in PBS was administered to the skin by the needle-free injection system.

All rabbits were vaccinated on days 0 and 14 of the experiment. Rabbits from Group 3 and Group 4 received an additional third booster vaccine on the 42nd day of the experiment. On days 28, 56, 75, and 105, blood was collected by ear vein sampling.

### 2.7. ELISA

For endpoint titer determination, a 96-well microtiter plate was coated with 2 µg/mL of RBD in 100 µL of PBS overnight at 4 °C. Afterward, the plate was blocked with 5% skimmed milk in PBS for 2 h at RT. Serial dilutions of mice/rabbit serum were added to the wells in 2.5% skimmed milk in PBS and incubated at RT for 60 min. Animal immune serum samples were heated at 55 °C for 30 min before use. After incubation, the wells were washed six times with 0.05% PBST and 100 µL of anti-IgG-HRP conjugated antibody (1:5000 dilution) was added. After 1 h of incubation and five washes, positive binders were determined upon 100 µL TMB substrate addition. The absorbance at 450 nm was determined using a Clariostar (BMG, Berlin, Germany) reader after stopping the reaction by adding 100 μL of 2M H_2_SO_4_ per well.

Endpoint titers were calculated using an in-house build Python script (https://github.com/MShapira?tab=overview&from=2022-09-01&to=2022-09-28; accessed on 1 July 2022). The algorithm allows the calculation of a cutoff value for each sample group and endpoint titer based on the estimated cutoff [47]. For the data approximation, a further equation is used:

(1) $$Y=\frac{y_{min}+y_{max}}{1+10^{\left(1+LogEC50-X\right)\times b}}$$

where:

*LogEC*50—the serum dilution that gives a response halfway between *y_min_* and *y_max_*;

*b*—the steepness of the curve.

This description of a four-parameter logistic curve allows the approximation of almost all experimental data, except for sets without at least one expressed plato (bottom or top) (Figure S1).

### 2.8. ELISPot

Spleens from immunized mice were collected in sterile tubes containing RPMI-1640 (ThermoFisher Scientific) media supplemented with 10% fetal bovine serum and 2X Antibiotic-Antimycotic (ThermoFisher Scientific). The cell suspension was obtained by rubbing the spleen through a 100 µm cell strainer (Corning, Corning, NY, USA). The mononuclear fraction of cells was separated from erythrocytes by gradient centrifugation on a Histopaque-1077 (Sigma-Aldrich, St. Louis, MO, USA).

Interferon gamma production was measured using the ELISpot Plus: Mouse IFN-γ (ALP) (Mabtech, Nacka Strand, Sweden). Splenocytes were counted and 250,000 cells were plated per well into pre-covered plates and stimulated overnight with 10 μg/mL SARS-CoV-2 (S1) peptide pool (Mabtech), medium as a negative control, or PHA 2 μg/mL as a positive experimental control. The next day, the plate was washed and treated with a biotinylated anti-IFN-γ detection antibody followed by a streptavidin–ALP conjugate, resulting in visible spots. The plates were dried and the spots were counted on an AID iSpot Spectrum (AID GmbH, Straßberg, Germany) and analyzed with EliSpot Software Version 7.x. Positive responses were defined as the number of spots/million cells in the test ≥2 SDEV above the negative control.

### 2.9. Statistical Analysis

The statistical analysis was performed using GraphPad Prism software 8.4 (GraphPad Software, Inc., LA Jolla, CA, USA).

## 3. Results

### 3.1. SARS-CoV-2 DNA Vaccine Candidates Design and In Vitro Analysis

A number of DNA-encoded vaccines have recently been developed, but all of them have the common drawback of insufficient levels of humoral responses in large animals [40,48,49].

We hypothesized that the oligomerization of DNA-encoded SARS-CoV-2 vaccine could enhance the humoral response, resulting in higher titers, even when using the same nucleic acid delivery technology and commonly used vectors. It also has been found that 97.9% of recovered COVID-19 patients exhibited high titer IgG specific for the HR region, but only the IgG titer to RBD was able to differentiate recurrent viral RNA-positive patients from persistently RNA-negative patients [50].

**Figure 1:**
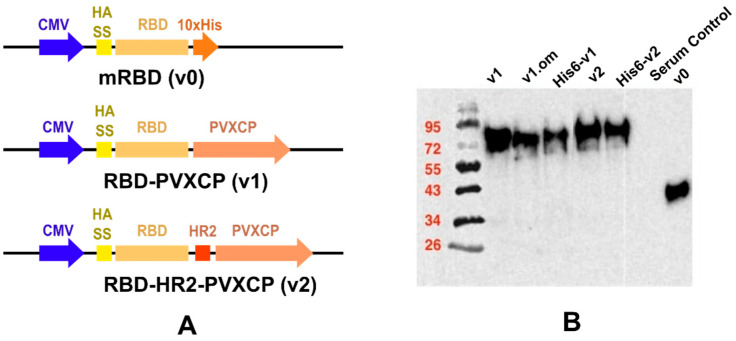
Schematic depiction of DNA vaccine constructs (**A**) and WB confirmation of secreted proteins in HEK293F culture supernatants (**B**).

We designed a number of DNA constructs (Figure 1A) encoding SARS-CoV-2 RBD proteins: (1) monomeric RBD (v0), (2) RBD fused with the PVXCP fragment via a rigid linker (RBD-PVXCP (v1)) and its variation with the N-terminal hexahistidine tag (His6-RBD-PVXCP (His6-v1)), (3) RBD-PVXCP protein with the HR2 region incorporated between them (RBD-HR2-PVXCP (v2)) and its variations with the N-terminal hexahistidine tag (His6-RBD-HR2-PVXCP (His6-v2)), and (4) RBD-PVXCP with the RBD fragment harboring Omicron B.1.1.529 mutations (oRBD-PVXCP (v1.om)).

**Figure 2:**
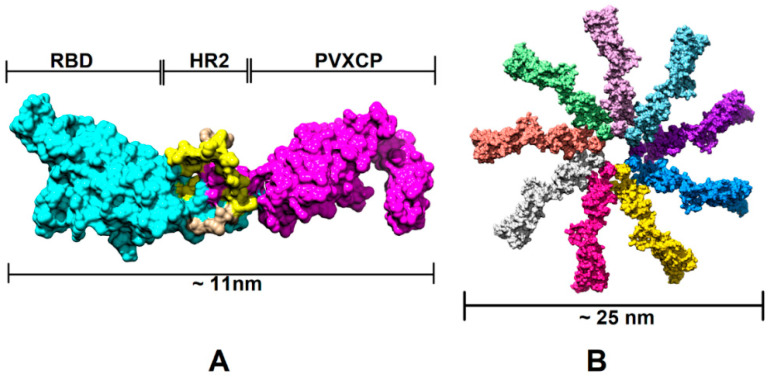
Structural illustration of the RBD-PVXCP vaccine based on the RBD (PDB: 7KLW) and PVXCP (PDB: 6R7G) structures (**A**) and its 9-mer structure (**B**). The illustrated size of the protein complex was determined in UCSF Chimera software.

In order to assess the RBD-HR2-PVXCP fusion vaccine protein and its behavior in a water solvent, 500 ns molecular dynamics (MD) simulation in the explicit solvent was carried out. Figure 2 illustrates the 3D structure of the RBD-HR2-PVXCP protein and its multimeric state after oligomerization, along with the expected diameter of the molecule.

Western blot analyses with the anti-S-protein polyclonal sera confirmed the soluble expression of vaccine candidates in the culture medium after the transient transfection of HEK293 FreeStyle (Figure 1B). The molecular weights of the proteins corresponded to those predicted theoretically, taking into account the glycosylation of RBD. The expression level of PVXCP fusion proteins was measured at 5 μg/mL, which was not inferior to the monomeric RBD transient transfection reported elsewhere [51].

In order to determine the RBD-PVXCP fusion protein oligomerization status, a hexahistidine (His-6) tag was added to the N-terminal parts of constructs v1 and v2 between the RBD and HA signal sequence. These fusion proteins were purified from 100 mL HEK293 FreeStyle supernatant by means of metal-chelating affinity chromatography (Ni-NTA) and were analyzed using dynamic light scattering (DLS). It was found that the majority of the proteins (>97% for RBD-PVXCP and >85% for RBD-HR2-PVXCP) had a radius of 3.5–12.7 nm (Figure S3). The weighted average radius of 11.3 nm for RBD-HR2-PVXCP corresponded to the size, ranging between a PVXCP disk (Figure 2B) and two PVXCP disks stacked together [52].

This result allows us to suggest the multimerization effect of PVXCP protein, in addition to its adjuvant properties. This also corresponds well to the generated molecular model of the RBD-HR2-PVXCP monomer and oligomer.

### 3.2. Humoral Response of DNA Vaccine Candidates in Mice

**Figure 3:**
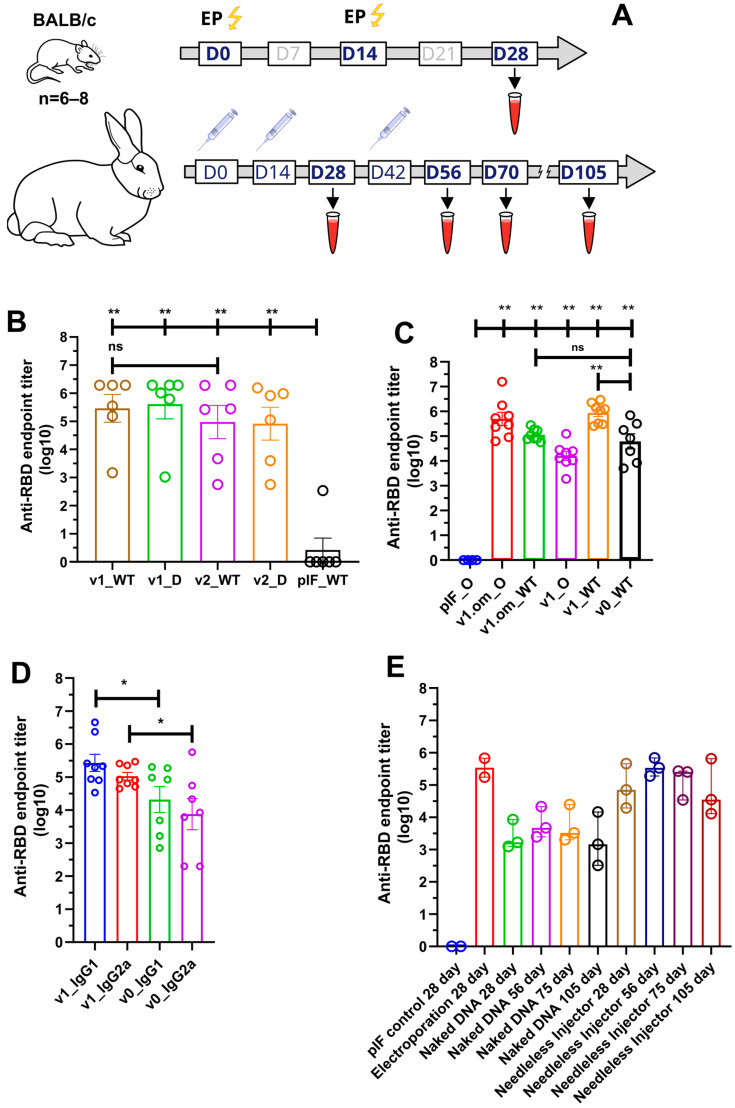
Analysis of RBD-specific humoral response. (**A**) Schematic representation of the experiment schedule; (**B**) RBD-specific IgG antibody titers from mice immunized with 2.5 μg of pDNA; (**C**) RBD (omicron and wild-type)-specific IgG antibody titers from mice immunized with 50 μg of pDNA; (**D**) Anti-RBD antibodies isotype analysis; (**E**) RBD-specific IgG antibody titers from rabbits immunized with 100 ug of pDNA by various routes; data presented as the mean ± SEM; ns—*p* value > 0.05, *—*p* values ≤ 0.05; **—*p* value < 0.01 (Mann–Whitney U-test).

The immunogenicity of the candidate DNA vaccines in mice was assessed in two experiments. First, we compared the RBD-specific IgG serum levels of mice immunized with DNA vaccine v1 or v2, or empty pIF vector. Three groups of female BALB/C mice (six animals per group) were immunized twice (with a 14-day interval) with 2.5 µg of pDNA, followed by electroporation at the injection site (Figure 3A). Two weeks after the booster immunization, blood was collected from all animals to evaluate the humoral response. All animals were sacrificed to obtain spleens for cellular response analysis.

Following immunization, no local inflammation responses at the injection site or other adverse effects were observed during the observation period. A primary/booster immunization of v1 and v2 induced the production of SARS-CoV-2 RBD-specific IgG antibodies, with the mean endpoint titer approaching 287,424 and 94,294, respectively, two weeks after the last immunization (Figure 3B). We also evaluated serum IgG binding to the B.1.617.2 (Delta) variant, with the resultant mean endpoint titers being 409,129 and 82,001 for v1 and v2, respectively.

In order to unleash the full antigenic potential of v1, a 50 µg pDNA dose was used for immunization, as well as the v1.om variant for the emerging B.1.1.529 SARS-CoV-2 variant. The adjuvant properties of PVXCP were assessed by comparing the humoral response to v1 and v1.om vaccines with the vaccine that encoded monomeric RBD (v0) without a fusion partner (Figure 3C). The calculated mean endpoint titers of the collected sera against Wuhan-Hu-1 RBD (WT) and B.1.1.529 (Omiron) are presented in Table 1.

Taking into account that ELISA antibody titers typically correlate with the neutralizing antibody titers, we utilized ELISA to compare the different designs’ IgG levels. It was found that the addition of PVXCP led to more than a tenfold increase in anti-RBD titers. Moreover, the binding of serum IgG from mice immunized with v1.om to wild-type RBD was only five times lower in comparison with the Omicron RBD binding, while the binding of IgG from mice immunized with v1 was fifty times lower for Omicron RBD in comparison with wild-type RBD. This suggests that the RBD-PVXCP vaccine with the RBD harboring Omicron mutations could be referred to as a more promising anti-SARS-CoV-2 vaccine candidate.

We determined the IgG subclasses of RBD-specific antibodies induced by v0 and v1 by sandwich ELISA (Figure 3D). Isotype analysis indicated that the binding antibodies elicited by monomeric RBD were IgG1 > IgG2a, indicating a strong Th2-dominant immune response [53]. The addition of PVXCP to the v1 design allowed the leveling of IgG1 and IgG2a antibodies, indicating a bias toward the more-preferred Th1 immune response.

### 3.3. A DNA Vaccine-Induced T Cell Response in Mice

**Figure 4:**
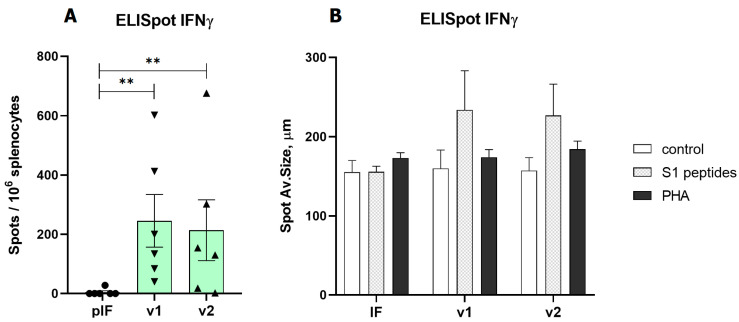
Analysis of RBD-specific cellular immune response to low-dose vaccines. (**A**)—Spot count for IFNγ, (**B**)—Spot size for IFNγ. pIF—vector control, v1—RBD-PVXCP vaccine, v2—RBD-HR2-PVXCP vaccine; data presented as the mean ± SEM; ** indicates the significance of differences (**—*p* value < 0.01) (Mann Whitney U-test).

In the first experiment (vaccination with 2.5 μg of pDNA), the T cell response was evaluated by ELISpot measurement of IFNγ production by splenocytes after S1 peptide pool stimulation. Both variants of v1 and v2 vaccines demonstrated a significant response. A similar pattern of vaccine immunogenicity was observed for the spot number and spot size in the ELISpot test (Figure 4).

**Figure 5:**
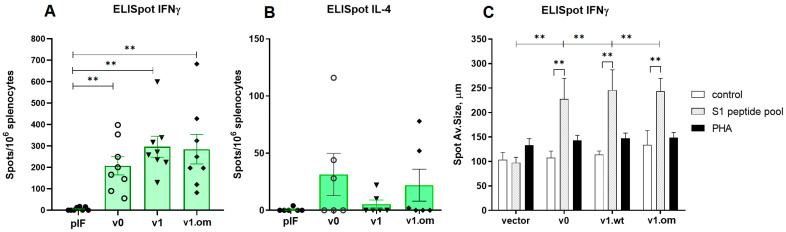
Analysis of RBD-specific cellular immune response to high-dose vaccines. (**A**) Spot count for IFNγ, (**B**) Spot count for IL-4, (**C**) Spot size for IFNγ; v0—monomer RBD (wt), v1—RBD (wt)-PVXCP, v1om—RBD (omicron)-PVXCP; data presented as the mean ± SEM; ** indicates the significance of the differences (*p* < 0.01) (Mann–Whitney U test).

In the second experiment with the 50 μg pDNA vaccines, the T cell response was estimated by ELISpot measurement of IFNγ and IL-4 production. The aim of the experiment was to compare the maximum immunogenicity potential of the v1 vaccine and v1.om against the RBD monomer (v0). The production of IFNγ in response to antigen stimulation in mice vaccinated with all three vaccine variants was significantly higher than that with the control vector, expressed both in spot count and average spot size (Figure 5A,C). The highest response was to v1, but the difference between vaccine variants was not significant (v0 vs. v1, *p* = 0.23). The calculations of the spot counts and immune response are specified in Table S1.

In each experimental group, there were 2–3 mice that responded to vaccination with IL-4 production, but the differences between vaccines and vector controls were not significant, and the intensity of the IL-4 response was significantly lower than that of IFNγ response (Figure 5B). The ratio IFNγ/IL-4 was 5.3 for v0 (*p* = 0.011), 48.3 for v1 (*p* < 0.001), and 8.9 for v1.om (*p* = 0.013).

### 3.4. Needle-Free Injection Delivery of RBD-PVXCP DNA Vaccine in Rabbits

We next evaluated the immunogenicity of the v1 anti-SARS-CoV-2 vaccine candidate in rabbits, an animal model well suited for immunological and toxicological studies [54]. As electroporation is a traumatic and painful procedure with very few apparatus options, and naked pDNA delivery in aqueous solutions lacks efficiency, we decided to explore a more robust and clinically tested approach—needle-free injection [55].

We compared three delivery routes for v1 pDNA delivery—EP (Group 2), needle injection of naked DNA (Group 3), and needle-free injection (Group 4). We performed primary/booster immunization in a two-week interval (day 0 and day 14) and the second booster for naked DNA and needle-free injection (day 56). The second booster immunization for electroporation was not performed due to some traumatic impact of this method on rabbits. The jet injection in Group 4 was well-tolerated and resulted only in a hematoma in some cases at the injection site that was almost fully recovered within 2 weeks after immunization. The needle injection (Groups 1 and 3) was well-tolerated and had no obvious local side effects.

The sera from vector control (pIF) Group 1 produced only the background level of the absorbance signal. The v1 pDNA vaccine induced an anti-RBD-specific immune response in all routes of administration. On day 28 (week 4), the mean RBD-specific end-point titers were 343,887, 2614, and 85,712 for EP, naked DNA, and needle-free injection respectively (Figure 3E). A second booster of naked DNA and needle-free injected DNA resulted in the mean endpoint titer increasing to 6274 and 352,906 at day 56. It is worth noting that, after the third immunization by needleless injection, the antibody titer matched the titer after electroporation in a primary/booster regimen.

Observation of Group 3 and Group 4 of rabbits after immunization was continued and two more blood samples (on day 75 and on day 105) were collected. The endpoint titer in Group 4 decreased to 132,882 on day 75 and to 66,633 on day 105.

## 4. Discussion

The COVID-19 pandemic is the first worldwide challenge of this scale since the H1N1 Spanish flu [56]. It greatly accelerated novel vaccines and therapeutic antibodies discovery, development, and deployment. Novel platforms were approved for clinical uses, including mRNA, DNA, and vector vaccines [57].

The global vaccine campaign also provides a unique opportunity to compare different platforms and technologies at an unprecedented scale. It has become apparent that mRNA and vector vaccines have surpassed traditional inactivated and recombinant protein vaccines in a term of efficiency [58]. Although more than 15 DNA vaccine candidates have entered different stages of clinical trials, only ZyCoV-D has been approved in a single country (https://covid19.trackvaccines.org/; accessed on 10 July 2022). Based on the results of a Phase III clinical trial, the ZyCoV-D SARS-CoV-2 DNA vaccine has been found to be 67% protective against symptomatic COVID-19 delivered by a needle-free injection device. India’s drug regulator has approved it as the first DNA vaccine for humans for prophylaxis against COVID-19. However, its efficiency is significantly lower than that of the approved mRNA vaccines Comirnaty and Spikevax, raising questions about the worldwide deployment of similar DNA vaccines [59,60].

Here, we approached SARS-CoV-2 DNA vaccine development with a rational design strategy of immunogen molecules. We developed two variants of the SARS-CoV-2 vaccine based on the fusion of RBD immunogen with the Potato virus X coat protein (PVXCP) adjuvant molecule in order to enhance the depth and quality of the immune response to RBD.

PVXCP could self-assemble into virus-like particles, even in the absence of Potato Virus X RNA, enhancing the depth of immune responses to cancer DNA vaccines [52]. Displaying antigens on nanoparticles or virus-like particles is an established strategy to increase immunogenicity. Multivalent antigens that can mimic the repetitive and well-ordered antigenic structures found on many pathogens can cross-link B-cell receptors and activate B-cells more efficiently than their monovalent counterparts. In addition, they can be taken up by antigen-presenting cells and trafficked to lymph nodes more efficiently, leading to the improved formation of germinal centers [61,62].

Different nanoparticle vaccines against SARS-CoV-2 have been developed and exhibit an advantage over their monovalent counterparts [63,64,65,66]. PVXCP could tolerate long polypeptides fused to its N-terminus, maintaining high protein expression. PVXCP has also been described as an immune enhancer that stimulates CD4 help and shifts the immune response toward Th1 [67].

Our data support the high level of the secretion of v1 and v2 vaccine candidates in the HEK293F system. It was revealed that PVXCP fused to the RBD with or without the HR2 region between them did not affect the RBD expression level. Purified RBD-PVXCP and RBD-HR2-PVXCP proteins encoded by v1 and v2 also tended to oligomerize in solution. This, if applicable under in vivo conditions, should result in a stronger humoral response in comparison with the monomeric RBD protein.

In mice, strong humoral and T cell responses were observed using 2.5 μg and 50 μg pDNA v1 and v2 electroporation. The anti-RBD IgG titers exceeded 105, even for the smaller 2.5 μg dose; furthermore, serum IgG binding to RBD was not affected by the presence of B.1.617.2 mutations in RBD. We also evaluated the humoral response elicited by the v1.om variant and compared it with those of v1 and v0. The addition of PVXCP to RBD elevated the RBD-specific IgG more than tenfold for the wild-type RBD. As a result, we observed immunogenicity that was not inferior, and sometimes superior, to the preclinical data of other DNA-based and mRNA-based vaccines [68,69,70].

Surprisingly, the omicron-based v1.om vaccine outperformed the wild-type-based v1 vaccine by eliciting more universal antibodies, which were more tolerant to RBD mutations.

We found a prominent T-cell immune response expressed in IFNγ production against the S1 peptide library in vaccinated mice. The highest response rates of 700 with a median of 200–300 spots per 10^6^ splenocytes were observed for both the 2.5 μg and 50 μg doses of the v1 and v2 vaccine candidates. This response rate exceeded the results of even the higher dose of the 100 μg DNA vaccine ZyCoV-D and approached the response rate of vaccines INO-4800 and pGO-1001/1002, which encode the whole Spike protein as an antigen [49,69,71]. In the same experiment, an immune response to the vaccine expressed in IL-4 production was observed in 33–50% of mice at a level several times lower than that of IFNγ production. However, the results of the T-cell response by the ELISpot method showed considerable scatter in the data and no significant difference between the different vaccine candidates. The v1 construct showed the best immunogenicity, but the data obtained in our experiments did not allow us to draw a definite conclusion about the better T-cell response of the v1, v1om, and v2 vaccines compared with v0.

The immunization of children with whole-inactivated-virus vaccines against RSV and measles virus led to vaccine-associated enhanced respiratory disease (VAERD), an immunopathological state associated with T helper 2 cell (TH2)-biased immune responses [72,73]. A similar phenomenon has been seen in some animal models with whole-inactivated vaccines, including SARS-CoV vaccines [74,75]. The significance of the Th1 cell response has also been shown for asymptomatic and mild forms of COVID-19 infections [76,77].

The higher IFN/IL-4 ratio in our experiments indicates the development of a Th1-type immune response in BALB/c mice naturally biased for Th2 immune response. This is presumably due to the role of the PVXCP protein, since the vaccine with the RBD monomer induced more apparent production of IL-4.

We also observed a PVXCP-induced bias towards Th1by comparing the isotypes of antibodies induced by the DNA-encoded RBD vaccine v0 and RBD-PVXCP v1. It was revealed that the IgG1>>IgG2a antibody levels for v0 shifted to the IgG1~Ig2a ratio for v1. This confirms our assumption that PVXPC could turn the anti-RBD immune response to a more favorable Th1 type.

Despite the long history of research on prophylactic and therapeutic DNA vaccines, DNA delivery is still a challenge. Electroporation is still the most frequent and effective way to deliver a DNA vaccine in preclinical animal trials. Several electroporation devices for humans have been developed, including MedPulser^®^ and Cellectra^®^ (Inovio Inc., Plymouth Meeting, PA, USA), TriGrid^®^ (Ichor Medical Systems, San Diego, CA, USA), and Cliniporator^®^ (IGEA medical, Carpi, Italy), including those intended for vaccination against SARS-CoV-2 [78]. However, electroporation devices are still too expensive, and the injection procedure is unpleasant for most patients for widespread prophylactic use.

Currently, needle-free injection is the most promising, affordable, and effective approach to delivering clinical-grade DNA vaccines via the intradermal or intramuscular route. PharmaJet (Golden, CO, USA) is a leading company in this field by providing its own needle-free injectors PharmaJet Tropis/Stratis to the market. ZyCoV-D, DIOSynVax, Nykode VB D-01, Scancell COVIDITY, and COVIGEN DNA vaccines are administered by means of PharmaJet needle-free injection systems.

Due to the possibility of sterilizing immunity acquisition after the administration of mucosal COVID-19 vaccines, there is a high rationale for the implementation of this route for DNA vaccine delivery. Formulating pDNA within liposomes, LNP, niosomes, or polymers (biodegradable PEI, chitosan derivates, PLGA, peptide–poloxamine nanoparticles, etc.) and different microorganisms (attenuated strains of *Salmonella*, bacteriophages, etc.) is a very perspective method of mucosal DNA vaccine delivery [79,80,81]. However, human efficacy data of mucosal DNA vaccines are still lacking and more clinical research is needed.

Therefore, we decided to compare the three-most-common methods of DNA delivery for our vaccine candidate—naked DNA injection, needle-free injection devices, and electroporation by means of immunizing large animals.

The RBD-PVXCP DNA vaccine also induced a strong humoral response in rabbits after primary/booster immunization. As electroporation has limited use in humans, especially for prophylactic vaccination, we focused on needle-free injection systems that are clinically approved, cheap, and deliver the payload with high efficiency without considerable side effects.

After the primary/booster set of our pDNAv1 vaccine, the mean RBD-specific end-point titer reached 85,712 utilizing the needle-free injection method. To the best of our knowledge, we obtained the highest IgG level among all the naked pDNA vaccines that were pre-clinically tested in rabbits without being non-applicable in human electroporation [40,49]. In order to further explore the potential of our vaccine candidate, in week 6, we performed a second booster that raised the mean endpoint titer to 352,906 approaching electroporation in the primary/booster regimen. Two months after the second booster, the IgG level was still high, with the end-point titer being 66,633. This confirms the long-lasting immunity induced by the RBD-PVXCP vaccine candidate.

Summarizing all of the above, DNA vaccine candidates that were rationally designed and developed in the present work demonstrated high immunogenicity and a good safety profile in two animal models—mice and rabbits. The evaluation of the neutralization potency of elicited antibodies and the prophylactic effect in SARS-CoV-2-challenged animal models is needed.

In conclusion, our current work demonstrates the feasibility and potency of SARS-CoV-2 DNA vaccines to induce anti-RBD IgG titer compared with mRNA-LNP vaccines, providing important information for the further development of DNA-based vaccines, even beyond SARS-CoV-2 infection.

## 5. Patents

DD, AMe, and MK have a pending patent application for the DNA-based SARS-CoV-2 vaccine.

## Figures and Tables

**Table 1:** RBD-specific IgG antibody mean endpoint titers from mice immunized with 50 µg of pDNA.

RBD/Vaccine	v0	v1	v1.om
WT	62,010	869,124	113,654
Omicron	NA	16,053	497,209

## Data Availability

The data presented in this study is available within the article or Supplementary Materials.

## Supplementary Materials

The following supporting information can be downloaded at: https://www.mdpi.com/article/10.3390/vaccines11061014/s1, Figure S1. Titer curves of the anti-RBD serum samples (A—mice immunization, B—rabbits immunization); Figure S2: Generic plasmid vector map for the pIF DNA vaccine delivery vector; Figure S3: Comparison of histograms of the DLS-measured particles’ distribution. Table S1. ELISpot spots count and response for each mouse.
