# Supplementary material for: Design and Immunogenicity of SARS-CoV-2 DNA Vaccine Encoding RBD-PVXCP Fusion Protein
Source: Vaccines (Basel). 2023 May 23;11(6):1014. doi: 10.3390/vaccines11061014 (PMC10300735; doi:10.3390/vaccines11061014)
Supplement: Supplementary file 1 [file vaccines-11-01014-s001.zip › vaccines-2235677-supplementary.pdf]

Design and Immunogenicity of SARS-CoV-2 DNA vaccine encoding RBD-PVXCP fusion protein

Supplementary Material

Figure S1. Titer curves of the anti-RBD serum samples (A – mice immunization, B – rabbits immunization).

A

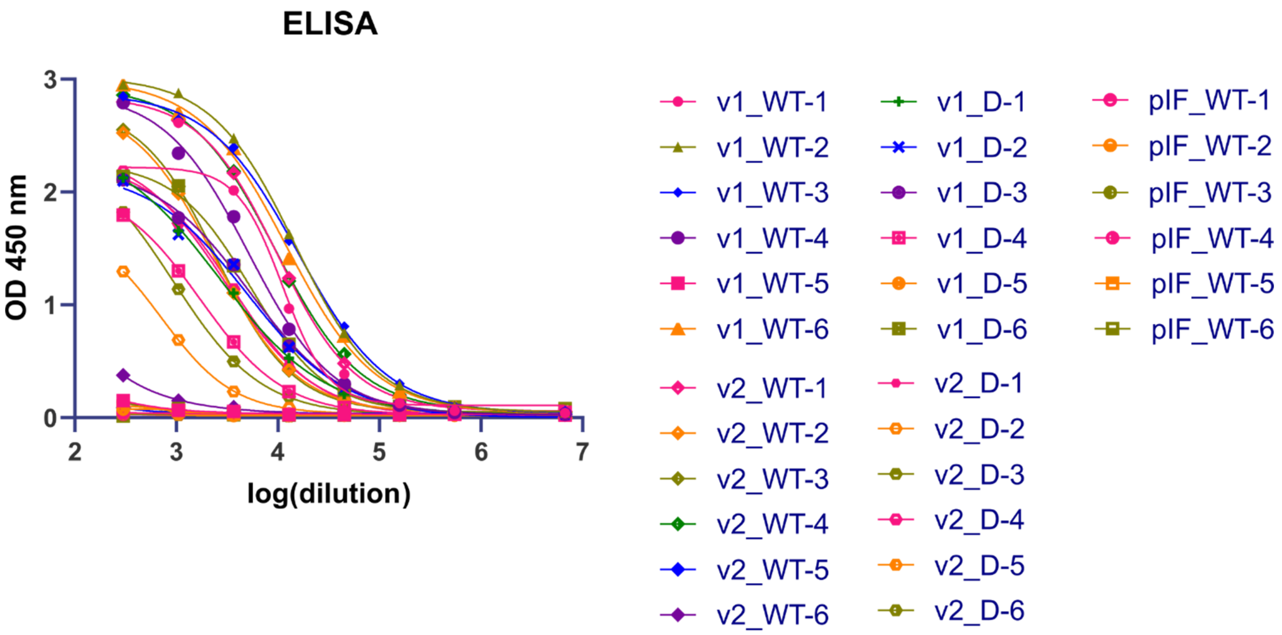

B

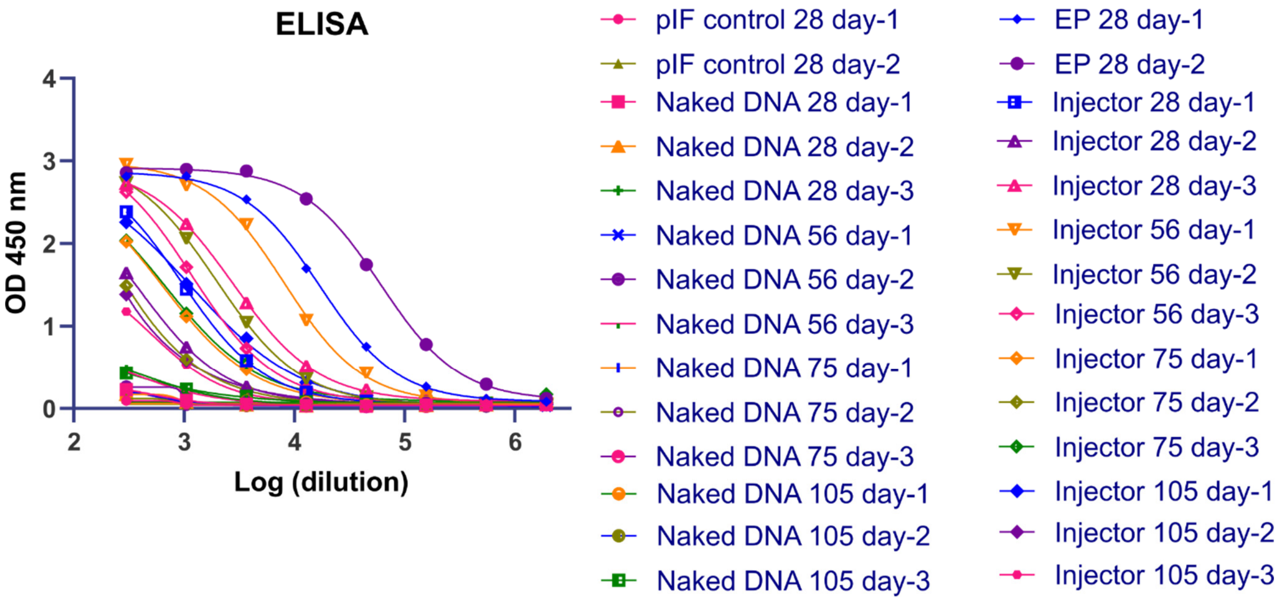

Design and Immunogenicity of SARS-CoV-2 DNA vaccine encoding RBD-PVXCP fusion protein

Figure S2. Generic plasmid vector map for the pIF DNA-vaccine delivery vector.

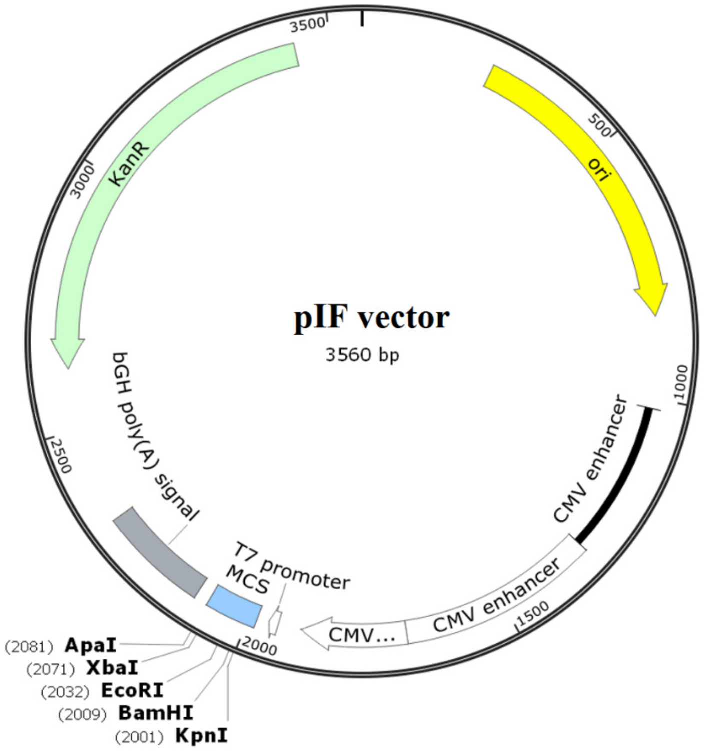

Figure S3. Comparing histograms of the DLS-measured particles distribution.

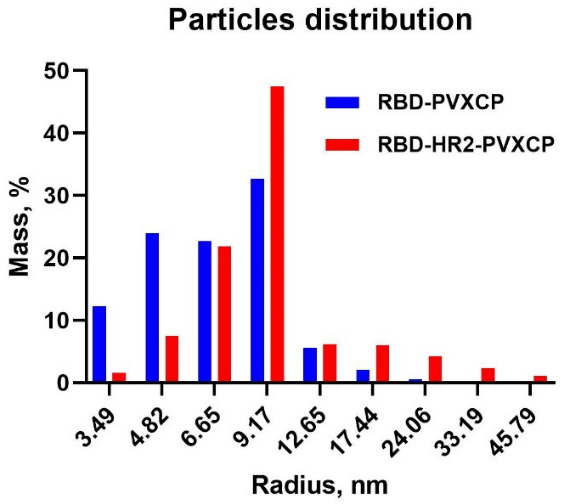

Design and Immunogenicity of SARS-CoV-2 DNA vaccine encoding RBD-PVXCP fusion protein

Table S1. ELISpot spots count and response for each mouse

S1A. ELISpot results for IFNγ in the first experiment (for figure 4)							
	Mean control	Mean test S1	test - control	SD	(test – control) - 2SD	Response	
Control_1	124	152	28	16.97	-5.94	negative	0/6
Control_2	118	104	-14	8.49	-30.97	negative	
Control_3	88	84	-4	5.66	-15.31	negative	
Control_4	198	164	-34	36.77	-107.54	negative	
Control_5	120	120	0	33.94	-67.88	negative	
Control_6	166	144	-22	8.49	-38.97	negative	
v1_1	138	550	412	8.49	395.03	positive	6/6
v1_2	202	804	602	31.11	539.77	positive	
v1_3	90	290	200	19.80	160.40	positive	
v1_4	92	176	84	5.66	72.69	positive	
v1_5	104	144	40	5.66	28.69	positive	
v1_6	268	402	134	11.31	111.37	positive	
v2_1	80	756	676	5.66	664.69	positive	4/6
v2_2	192	346	154	11.31	131.37	positive	
v2_3	132	262	130	16.97	96.06	positive	
v2_4	282	584	302	2.83	296.34	positive	
v2_5	300	318	18	33.94	-49.88	negative	
v2_6	190	192	2	53.74	-105.48	negative	
**S1B. ELISpot results for IFNγ in the second experiment (for figure 5)**							
	Mean control	Mean test S1	test - control	SD	(test – control) - 2SD	Response	
Control_1	325.0	341.0	16.0	33.94	-51.84	negative	0/8
Control_2	289.3	217.0	-72.3	16.97	-106.26	negative	
Control_3	397.9	285.0	-112.8	11.31	-135.45	negative	
Control_4	297.8	245.7	-52.1	5.66	-63.43	negative	
Control_5	470.2	414.3	-55.9	36.77	-129.48	negative	
Control_6	177.7	194.9	17.2	11.30	-5.427	negative	
Control_7	295.5	308.1	12.6	14.14	-15.71	negative	
Control_8	186.6	131.3	-55.3	50.91	-157.12	negative	
v0_1	181.4	327.5	146.1	11.31	123.49	positive	8/8
v0_2	156.0	246.1	90.0	0.00	90.02	positive	
v0_3	131.8	187.1	55.2	11.31	32.60	positive	
v0_4	140.5	341.0	200.5	0.00	200.48	positive	
v0_5	141.1	539.4	398.3	14.14	369.98	positive	
v0_6	204.8	370.6	165.8	25.46	114.89	positive	
v0_7	93.3	447.4	354.1	67.88	218.33	positive	
v0_8	228.0	476.9	248.9	42.43	164.05	positive	
v1_1	253.8	489.2	235.4	14.14	207.16	positive	8/8

**v1_2**	174.8	513.4	338.6	11.31	315.97	positive	
**v1_3**	114.0	337.3	223.3	28.28	166.70	positive	
**v1_4**	67.8	384.5	316.6	0.00	316.62	positive	
**v1_5**	184.1	441.9	257.8	2.83	252.14	positive	
**v1_6**	93.2	222.3	129.1	2.83	123.43	positive	
**v1_7**	156.8	755.8	599.1	48.08	502.90	positive	
**v1_8**	138.7	409.8	271.1	0.00	271.12	positive	
**v1om_1**	236.8	919.2	682.4	56.57	569.29	positive	**8/8**
**v1om_2**	122.6	319.1	196.5	11.31	173.85	positive	
**v1om_3**	155.1	237.2	82.1	11.31	59.49	positive	
**v1om_4**	113.4	233.4	120.0	16.97	86.03	positive	
**v1om_5**	267.2	516.6	249.4	16.97	215.46	positive	
**v1om_6**	101.1	528.6	427.5	16.97	393.60	positive	
**v1om_7**	142.9	340.0	197.0	42.43	112.18	positive	
**v1om_8**	104.5	429.2	324.7	2.83	319.03	positive	

S1C. ELISpot results for IL-4 in the second experiment (for figure 5)

	**Mean control**	**Mean test S1**	**test - control**	**SD**	**(test – control) - 2SD**	**Response**	
**Control_1**	212.0	208.0	-4.0	5.66	-15.31	negative	**0/6**
**Control_2**	118.0	78.0	-40.0	8.49	-56.97	negative	
**Control_3**	70.0	74.0	4.0	19.80	-35.60	negative	
**Control_4**	102.0	52.0	-50.0	25.46	-100.91	negative	
**Control_5**	194.0	186.0	-8.0	59.40	-126.79	negative	
**Control_6**	142.0	134.0	-8.0	19.80	-47.60	negative	
**v0_1**	220.0	264.0	44.0	0.00	44.00	positive	**3/6**
**v0_2**	118.0	112.0	-6.0	31.11	-68.23	negative	
**v0_3**	136.0	136.0	0.0	50.91	-101.82	negative	
**v0_4**	160.0	148.0	-12.0	11.31	-34.63	negative	
**v0_5**	114.0	230.0	116.0	48.08	19.83	positive	
**v0_6**	58.0	86.0	28.0	2.83	22.34	positive	
**v1_1**	74.0	96.0	22.0	31.11	-40.23	negative	**0/6**
**v1_2**	182.0	170.0	-12.0	36.77	-85.54	negative	
**v1_3**	86.0	96.0	10.0	36.77	-63.54	negative	
**v1_4**	182.0	166.0	-16.0	2.83	-21.66	negative	
**v1_5**	96.0	96.0	0.0	22.63	-45.25	negative	
**v1_6**	238.0	192.0	-46.0	87.68	-221.36	negative	
**v1om_1**	234.0	312.0	78.0	15.55	46.88	positive	**2/6**
**v1om_2**	450.0	502.0	52.0	8.49	35.03	positive	
**v1om_3**	226.0	152.0	-74.0	25.46	-124.91	negative	
**v1om_4**	30.0	30.0	0.0	19.80	-39.60	negative	
**v1om_5**	264.0	204.0	-60.0	11.31	-82.63	negative	
**v1om_6**	74.0	76.0	2.0	48.08	-94.17	negative	
